# Uso de psicofármacos entre comunidades indígenas y quilombolas en Brasil

**DOI:** 10.18294/sc.2024.4892

**Published:** 2024-12-11

**Authors:** Gabriela da Silva Gonçalves Loures, Telmo Mota Ronzani, Patrícia Aparecida Baumgratz de Paula, Magda Dimenstein, Saulo Luders Fernandes, Jáder Ferreira Leite, João Paulo Sales Macedo, Gabriel Martins Silva

**Affiliations:** 1 Magíster en Salud Colectiva. Estudiante del Doctorado en Salud Colectiva, Universidade Federal de Juiz de Fora, Minas Gerais, Brasil. gabrielamef@gmail.br Universidade Federal de Juiz de Fora Universidade Federal de Juiz de Fora Minas Gerais Brasil gabrielamef@gmail.br; 2 Doctor en Ciencias de la Salud. Profesor titular, Universidade Federal de Juiz de Fora, Minas Gerais, Brasil. tm.ronzani@gmail.com Universidade Federal de Juiz de Fora Universidade Federal de Juiz de Fora Minas Gerais Brasil tm.ronzani@gmail.com; 3 Doctora en Salud Colectiva. Profesora titular, Universidade Federal de Juiz de Fora, Campus Governador Valadares, Minas Gerais, Brasil. patricia.paula@ufjf.br Universidade Federal de Juiz de Fora Universidade Federal de Juiz de Fora Campus Governador Valadares Minas Gerais Brasil patricia.paula@ufjf.br; 4 Doctora en Salud Mental. Profesora titular, Universidad Federal de Río Grande del Norte, Rio Grande do Norte, Brasil. magda.dimenstein@ufrn.br Universidade Federal do Rio Grande do Norte Universidad Federal de Río Grande del Norte Rio Grande do Norte Brasil magda.dimenstein@ufrn.br; 5 Doctor en Psicología Social. Profesor asociado, Universidade Federal de Alagoas, Alagoas, Brasil. saulo@ip.ufal.br Universidade Federal de Alagoas Universidade Federal de Alagoas Alagoas Brasil saulo@ip.ufal.br; 6 Doctor en Psicología Social. Profesor asociado, Universidad Federal de Río Grande del Norte, Rio Grande do Norte, Brasil. jader.leite@ufrn.br Universidad Federal de Río Grande del Norte Universidad Federal de Río Grande del Norte Rio Grande do Norte Brasil jader.leite@ufrn.br; 7 Doctor en Psicología. Profesor titular, Universidade Federal do Delta do Parnaíba, Parnaíba, Brasil. jampamacedo@gmail.com Universidade Federal da Paraíba Universidade Federal do Delta do Parnaíba Parnaíba Brasil jampamacedo@gmail.com; 8 Estudiante de Psicología, Universidade Federal de Juiz de Fora, Minas Gerais, Brasil. martinssilvagabriel@gmail.com Universidade Federal de Juiz de Fora Universidade Federal de Juiz de Fora Minas Gerais Brasil martinssilvagabriel@gmail.com

**Keywords:** Pueblos Indígenas, Quilombola, Psicofármacos, Medicalización, Salud Mental, Brasil, Indigenous Peoples, Quilombola Communities, Psychotropics Drugs, Medicalization, Mental Health, Brazil

## Abstract

El uso de psicofármacos es hoy un problema de salud pública debido al elevado consumo a nivel mundial. En el contexto de las poblaciones tradicionales, la bibliografía sobre el uso de estos medicamentos es escasa, por lo que se realizó un estudio descriptivo sobre el uso de psicofármacos en comunidades indígenas y quilombolas de los estados de Minas Gerais, Rio Grande do Norte, Alagoas y Piauí en Brasil. La recolección de datos se realizó entre marzo y junio de 2023, para lo cual se diseñaron dos cuestionarios estructurados con preguntas cerradas para ser aplicados en las visitas domiciliarias, en los que se relevó el perfil sociodemográfico de las comunidades indígenas y quilombolas y el uso general de psicofármacos. De las 335 personas de los cuatro estados, que respondieron los cuestionarios sobre el uso general de psicofármacos, 53 personas consumían psicofármacos, predominantemente mujeres adultas, con pocos años de escolaridad, de mayor edad, de bajos ingresos y dependientes de programas sociales y la clase terapéutica más utilizada fueron los antidepresivos. Se concluye que la fragilidad económica y social es probablemente un factor importante en la determinación social del malestar psicológico y el uso de psicofármacos.

## Abstract (en)

The use of psychotropic drugs has become a public health issue due to their high consumption worldwide. In the context of traditional populations, the literature on the use of these medications is scarce. This study presents a descriptive analysis of psychotropic drug use among Indigenous and Quilombola communities in the states of Minas Gerais, Rio Grande do Norte, Alagoas, and Piauí in Brazil. Data collection was conducted between March and June 2023 using two structured questionnaires with closed-ended questions applied during home visits. These questionnaires gathered sociodemographic profiles of the Indigenous and Quilombola communities and general psychotropic drug usage. Out of 335 individuals from the four states who responded to the questionnaires on general psychotropic drug use, 53 reported using these medications. The users were predominantly adult women with low educational attainment, older age, low income, and reliance on social programs. Antidepressants were the most commonly used therapeutic class. The study concludes that economic and social vulnerability is likely a significant factor in the social determinants of psychological distress and psychotropic drug use.

## INTRODUCCIÓN

El uso de psicofármacos es tratado actualmente como una cuestión de salud pública debido a los altos índices de consumo a nivel mundial, ya sea por prescripción excesiva o por automedicación, y por tratarse de medicamentos capaces de causar dependencia física, psíquica y otros efectos adversos. Según Barros y Silva^1^, el número de personas que consumen al menos un medicamento psicotrópico está en aumento, al igual que la prescripción y venta en Brasil de antipsicóticos y ansiolíticos benzodiazepínicos. 

Datos de la Pesquisa Nacional sobre Acesso, Utilização e Promoção do Uso Racional de Medicamentos no Brasil (PNAUM) indican que entre los 20 subgrupos farmacológicos más utilizados por los usuarios de la red básica de salud se encuentran los antidepresivos (fluoxetina), antiepilépticos y ansiolíticos (clonazepam)^2^. De esta manera, para Barros y Silva:

…el análisis del perfil de consumo de medicamentos psicofármacos se torna esencial para el monitoreo y perfeccionamiento de las políticas de acceso y el cuidado a los usuarios, contribuyendo a la mejora de las condiciones de salud mental de la población brasileña y el uso racional de los medicamentos.^1^

La bibliografía especializada señala una mayor prevalencia en el uso de psicofármacos por parte de las mujeres^3^, en grupos etarios de entre 40 y 59 años^4^ y entre los usuarios del primer nivel de atención^5^. Destaca que el aumento en la utilización de psicofármacos actualmente involucra desde múltiples y complejos determinantes macroestructurales relativos a las condiciones de vida y desigualdades sociales, hasta factores coyunturales, como la creación de nuevos diagnósticos, una mayor frecuencia de diagnósticos de trastornos psiquiátricos entre adultos, niños y adolescentes, así como la introducción de nuevos medicamentos en el mercado farmacéutico. 

Este fenómeno se convierte en un negocio muy lucrativo que se sostiene, en parte, en lo que Secco y Tesser señalan como la “ilusión social de la solidez de los beneficios del tratamiento psicofarmacológico”^6^. Sin embargo, de acuerdo a estos autores, existe cierta unanimidad en la bibliografía respecto a la asociación de la “progresiva masificación del uso de psicofármacos y la creciente medicalización de las vivencias y los sufrimientos de la vida”^6^. En este sentido, cobra relevancia la fuerza de la racionalidad biomédica en la gestión de la vida. No es casualidad que la industria farmacéutica y el cuerpo médico se configuren como agentes decisivos en la inducción al consumo de psicofármacos, cuya función es apaciguar el sufrimiento, la angustia y el malestar experimentados por las personas en su cotidianidad, en un intento por alcanzar los ideales de normalidad, bienestar, felicidad y éxito establecidos socialmente. 

Por esta razón, el concepto de medicalización de la vida, junto con el de patologización de la cotidianidad, se convierten en operadores conceptuales importantes para explicar la “epidemia de drogas psiquiátricas” y el “fetichismo de los psicofármacos” observados en las sociedades capitalistas contemporáneas^7^. Ambos conceptos se refieren a la invasión de la racionalidad biomédica en todos los sectores de la vida y a la transformación de problemas sociales, adversidades cotidianas, ciertos estilos de vida y comportamientos no deseados en cuestiones de salud o problemas médicos susceptibles de intervención y tratamiento. La medicalización de la existencia está vinculada a la patologización de las formas de vivir que escapan de lo deseado socialmente y que son consideradas anormales. En este sentido, los diagnósticos y medicamentos son vistos como recursos imprescindibles para la conservación y gestión de la “salud”, la prevención de riesgos, el entumecimiento y el control de los individuos. 

Cuando transponemos esta problemática a las poblaciones tradicionales, el panorama se vuelve bastante incierto, con muchas lagunas en la bibliografía sobre el uso de psicofármacos por parte de indígenas y quilombolas, lo que impide la detección y dimensión del problema para la formulación de una estrategia culturalmente adecuada. El desconocimiento de esta realidad forma parte de un conjunto de mecanismos que contribuyen a ampliar la marginación social de estas poblaciones, afirmando existencias pobres y precarias. En Brasil, son cada vez más frecuentes la fragilización y el desmantelamiento de los derechos constitucionales de poblaciones y comunidades tradicionales, así como las prácticas de racismo, la violencia y el exterminio. Los ataques a sus tierras y comunidades, los conflictos violentos y el retroceso de los derechos básicos se reflejan en una pérdida considerable de años y de calidad de vida, y en el aumento de la prevalencia de trastornos mentales^8^.

Como afirman Batista y Zanello^9^ en su estudio de revisión sistemática sobre la salud mental en contextos indígenas, que abarca de 1999 a 2012, se identificaron únicamente 14 artículos publicados en ese período. Además de ser incipiente, las publicaciones analizadas no debatían críticamente las definiciones de salud mental ni abarcaban la complejidad de las cosmologías y las comprensiones de los modos de vida indígena. Estas problemáticas teóricas y epistemológicas dificultan el diálogo con la diversidad de realidades de los pueblos indígenas de Brasil.

Las emergencias humanitarias que afectan a las poblaciones más vulnerabilizadas, incluidas las poblaciones tradicionales, han cobrado su precio no solo en las estadísticas de morbimortalidad, sino también en la salud mental, como ocurrió durante la pandemia de covid-19 y en los casos de rompimientos criminales de algunas represas relacionadas con la minería en el país^10^^,^^11^^,^^12^. No es casualidad que el aumento de casos de suicidio, el consumo abusivo de alcohol y sustancias psicoactivas entre indígenas sea una realidad observada desde la década de 1990^13^^,^^14^^,^^15^. Este aumento está relacionado con las violencias vividas en los contextos de los conflictos indígenas en Brasil, como señala el estudio de Barbosa, Cabral y Alexandre^13^, que investigó el uso de psicofármacos en el territorio Xukuru do Ororubá tras el conflicto ocurrido en la región en 2003. El estudio identificó que la población adulta del territorio se encontraba vulnerable desde el punto de vista socioeconómico, con un patrón crónico en el consumo de psicofármacos y una fragilización de las prácticas tradicionales de cuidado indígenas, resultado de la medicalización de la salud sobre el pueblo Xukuru.

En cuanto a los quilombolas, la bibliografía apunta a un aumento de la prevalencia de trastornos mentales, catalizados principalmente por la desigualdad social, el prejuicio y el racismo^16^^,^^17^^,^^18^^,^^19^. La revisión sistemática desarrollada por Batista y Rocha^20^, que realizó una búsqueda exhaustiva sin limitación de años de publicación sobre el tema salud mental y quilombos, identificó únicamente 18 artículos publicados. Los artículos analizados señalan que las desigualdades sociales, la vulneración de derechos y la falta de acceso a los servicios de salud experimentados por la población quilombola son los principales factores de deterioro de la salud mental en los territorios.

Ante la carencia de investigaciones dirigidas a las poblaciones tradicionales, en lo que respecta a la incidencia de trastornos mentales, el uso de alcohol y otras drogas, y el consumo de psicofármacos, además de la evidente dificultad para realizar investigaciones con poblaciones vulnerables y de difícil acceso debido a sus especificidades culturales, el objetivo de este estudio es analizar el perfil del uso de psicofármacos en comunidades indígenas y quilombolas de los estados de Minas Gerais, Rio Grande do Norte, Alagoas y Piauí en Brasil.

## MÉTODOS

Se trata de un estudio transversal y descriptivo, que busca conocer el perfil de uso de psicofármacos en comunidades indígenas y quilombolas de Minas Gerais, Rio Grande do Norte, Alagoas y Piauí^21^. Forma parte del proyecto multicéntrico “*Saúde Mental e Povos Tradicionais: desenvolvimento de tecnologias de detecção, análise e atenção para populações vulneráveis de difícil acesso*”, resultado de la colaboración entre la Universidade Federal de Juiz de Fora (UFJF), la Universidade Federal do Rio Grande do Norte (UFRN), la Universidade Federal de Alagoas (UFAL) y la Universidade Federal do Delta do Parnaíba (UFDPAR).

### Participantes y comunidades

El criterio para la selección de las comunidades fue la proximidad con la sede de las universidades participantes y la presencia concomitante de los dos tipos de poblaciones en el mismo municipio. En Minas Gerais, participaron el Quilombo dos Candendês en Ponto Chique do Martelo y las familias indígenas que habitaban en el distrito de Padre Brito, ambas situadas en el municipio de Barbacena. En Alagoas se seleccionaron la comunidad quilombola Tabacaria y la aldea indígena Xucuru Kariri Mata da Cafurna, ambas en el municipio de Palmeira dos Índios. En el caso de Rio Grande do Norte, formó parte de la investigación únicamente la comunidad indígena Tapará, situada en el municipio de Macaíba, dado que las lideranzas de las comunidades quilombolas no otorgaron autorización. Por último, en Piauí, se incluyó la comunidad quilombola Sussuarana y la comunidad indígena Canto da Várzea, ambas ubicadas en el municipio de Piripiri. 

Se entrevistó al mayor número posible de residentes mayores de 18 años que habitaban en las comunidades mencionadas y que dieron su consentimiento para responder los instrumentos utilizados en la recolección de los datos. No fue posible estimar el numero total de residentes por no haber datos oficiales en las comunidades participantes.

### Instrumentos

Para la recolección de datos se utilizaron cuestionarios estandarizados con preguntas cerradas, aplicados por los investigadores en forma de entrevista, en el marco de las visitas domiciliarias a las comunidades. La recolección se llevó a cabo durante el primer semestre de 2023.

El primer instrumento utilizado fue un cuestionario estructurado para la caracterización socioeconómica tanto de la persona encuestada (sexo, ocupación, nivel educativo, estado civil, raza/etnia, religión), como de su familia. Este se subdividió en las siguientes secciones: infraestructura y aspectos sanitarios, atención a la salud, asistencia social, y programas de crédito. Este cuestionario se aplicó por vivienda y lo respondió la persona responsable de la familia, considerada un informante clave que proporcionó información sobre su núcleo familiar (229 familias).

El segundo instrumento fue un cuestionario aplicado de forma individual a todas las personas residentes mayores de 18 años que aceptaron participar del estudio. Es decir, además de los informantes claves de cada familia, se administró un segundo cuestionario con seis preguntas orientadas al perfil farmacoterapéutico de los participantes que declararon utilizar psicofármacos, ya sean prescritos o no, así como otros tipos de compuestos como tés, jarabes, infusiones, entre otros. La recolección arrojó un total de 335 personas, de las cuales 80 utilizaban algún compuesto (tés, infusiones, entre otros), y 53 usaban psicofármacos. A través de estas preguntas fue posible delinear un perfil del uso de psicofármacos en las comunidades. Este instrumento es una adaptación del método de Morisky-Green-Levine, validado en portugués, con cuatro preguntas, utilizado para verificar la adherencia a la farmacoterapia^22^, y también evalúa la forma de acceso a los medicamentos.

### Análisis de datos

La base de datos fue organizada y digitalizada en el software estadístico Statistical Package for the Social Sciences (SPSS), versión 15. En un primer momento se delineó el perfil sociodemográfico y socioeconómico de las familias de las comunidades, la expresión de los datos de frecuencia, y para la edad de los participantes se calcularon la media y la desviación estándar. También se calculó la prevalencia del uso general de psicofármacos con relación al perfil sociodemográfico y socioeconómico de las comunidades.

### Aspectos éticos

Todo el proceso de investigación fue presentado y discutido con los líderes de cada comunidad, y la investigación solo se llevó a cabo con autorización previa. Además, se asumió el compromiso de presentar los resultados a los miembros de la comunidad y a los gestores, con el objetivo de colaborar en la mejora de las condiciones de salud de las comunidades.

A cada participante se le presentó el consentimiento libre e informado (TCLE), y se les garantizó total confidencialidad de los datos. Se utilizó un sistema de codificación para no revelar la identidad de las personas que aceptaron proporcionar su información. También se aseguró un ambiente aislado para la realización de las entrevistas, con el fin de preservar la privacidad de la persona participante. El proyecto fue sometido al Consejo Nacional de Ética en Investigación (CONEP) y aprobado mediante el dictamen 4.669.056.

## RESULTADOS Y DISCUSIÓN

Participaron en el estudio 229 familias distribuidas en los cuatro estados: Alagoas (24,0%), Minas Gerais (27,5%), Piauí (40,6%) y Rio Grande do Norte (7,9%). En cuanto a la pertenencia étnica/racial, el 59,4% de las familias eran quilombolas, mientras que el 40,6% eran indígenas. Se trata de personas que llevaban mucho tiempo residiendo en el territorio: el 41,5% vivía en los lugares investigados desde la formación de la comunidad y su nacimiento, y el 44,1% habitaba en las comunidades desde hacía más de seis años. La mayoría de estas familias (51,5%) tenían entre 3 y 5 integrantes, pero también era significativa la proporción de familias formadas por hasta 2 personas (37,6%).

En términos del perfil socioeconómico, el 68,7% de las familias sobrevivían con hasta un salario mínimo, y las principales fuentes de ingresos eran la agricultura familiar (22,7%), las parcelas de cultivo (32,3%), el trabajo asalariado (24,0%) y la jubilación (28,8%). Un dato particularmente importante es que el 44,5% del total de familias declaró que los programas sociales forman parte importante de su presupuesto familiar.

En cuanto al saneamiento, el 72,5% de los domicilios no tenía acceso a una red pública de agua, recurriendo al uso de agua proveniente de pozos, estanques, cisternas o cedida. Respecto a la calidad del agua consumida, el 69,0% de estas familias utilizaba agua tratada, ya sea mediante cloración, ebullición o filtración, mientras que el 28,0% declaró consumir agua no tratada. Predominaba el uso de fosas sépticas como solución para la disposición de desechos en el 74,2% de las viviendas. En cuanto al acceso a la energía eléctrica, el 97% de las casas disponía de este recurso.

Según la información aportada por los informantes claves de cada una de las familias (n=229), el 34,9% (n=80) tenía algún miembro que usaba psicofármacos. Entre las 335 personas entrevistadas individualmente, el 15,8% (n=53 personas) era usuaria de psicofármacos en los cuatro estados, la mayoría de ellas residentes en Minas Gerais y Piauí. Con relación a la edad de las personas entrevistadas, la media fue de 48,4 años, con una desviación estándar de 16,38 años. La Tabla 1 muestra un predominio del uso de psicofármacos entre mujeres adultas, con pocos años de escolaridad, mayor edad, bajos ingresos y dependientes de programas sociales. Independientemente de pertenecer a una comunidad indígena o quilombola, los datos corroboran los estudios que señalan una prevalencia de género y otras características sociales en el consumo de psicofármacos^3^^,^^23^. Además, destacan la fragilidad económica y las precarias condiciones de vida de estos territorios, que probablemente actúan como importantes factores de determinación social del sufrimiento psíquico y del uso de psicofármacos^15^^,^^24^.

**Tabla 1:** Perfil sociodemográfico y socioeconómico de la población estudiada y de las personas usuarias de psicofármacos en comunidades indígenas y quilombolas. Estados de Minas Gerais, Rio Grande do Norte, Alagoas y Piauí, Brasil, marzo a junio de 2023.

Variables	Población estudiada (n=335)		Personas usuarias de psicofármacos (n=53)	
	n	%	n	%
**Sexo**				
Femenino	211	66,0	41	77,4
Masculino	124	37,0	12	22,6
**Estado civil**				
No casado	137	40,9	21	39,6
Casado	198	59,1	32	60,4
**Raza/color**				
Negra	204	61,1	28	52,8
Indígena	121	36,2	22	41,5
Blanca	9	2,7	3	5,7
Comunidad				
Indígena	133	39,7	27	50,9
Quilombola	202	60,3	26	49,1
**Tiempo que reside en el territorio**				
Hasta 6 años	43	12,9	3	5,7
Más de 6 años	291	87,1	50	94,3
**Estados**				
Minas Gerais	93	27,8	24	45,3
Alagoas	60	17,9	9	17,0
Rio Grande do Norte	25	7,5	5	9,4
Piauí	157	46,9	15	28,3
**Ingresos**^1^				
Hasta 1 salario mínimo	218	65,1	32	60,4
Más de 1 salario mínimo	115	34,3	21	39,6
**Escolaridad**^2^				
Hasta enseñanza primaria completa	222	66,3	35	66,0
Enseñanza primaria completa y más	110	32,8	17	32,1
**Acceso a programas de asistencia social**				
Sí	257	76,7	45	84,9
No	78	23,8	8	15,1
**Acceso a programas de crédito**				
Sí	226	67,5	24	45,3
No	109	32,5	29	54,7
**Número de personas por vivienda**				
Hasta 2 personas	113	33,7	16	30,2
Más de 2 perdonas	222	66,3	37	69,8

Fuente: Elaboración propia. ^1^En la variable ingresos, en población estudiada, dos personas no respondieron. ^2^En la variable escolaridad, en población estudiada, tres personas no respondieron y en personas usuarias de psicofármacos, una persona no respondió.

En cuanto al perfil de acceso a la salud, los servicios de atención primaria de salud (APS), como la unidad básica de salud y el puesto de salud, son los principales puntos de acceso, la mayoría ubicados en la propia comunidad (69,9%), lo que indica una oferta de servicios cercana a los territorios habitados por estas poblaciones. En el ámbito de la APS, los agentes comunitarios de salud (84,5%) representan la principal estrategia utilizada por los residentes, lo que sugiere que su presencia en los territorios quilombolas e indígenas facilita la creación de vínculos con la comunidad y la identificación de sus necesidades en salud. Se observa, sin embargo, una limitada presencia de acceso a los equipos de la Estrategia de Salud de la Familia (ESF) y del Núcleo Ampliado de Salud de la Familia (NASF) por parte de los residentes, lo que podría impactar en la detección temprana de problemas asociados al uso de psicofármacos y a la salud mental en general (Tabla 2).

**Tabla 2:** Acceso a los servicios de salud por parte de la población estudiada y de las personas usuarias de psicofármacos en comunidades indígenas y quilombolas. Estados de Minas Gerais, Rio Grande do Norte, Alagoas y Piauí, Brasil, marzo a junio de 2023.

Variables	Población estudiada (n=335)		Personas usuarias de psicofármacos (n=53)	
	n	%	n	%
Acceso a la atención de salud*				
Agentes comunitarios de salud	283	84,5	46	86,8
ESF/ NASF	65	19,4	19	35,8
Puesto de Salud	162	48,4	18	33,9
Unidad básica de salud	86	25,7	17	32,1
Hospital/Emergencia	111	33,1	17	32,1
Ubicación del establecimiento de salud				
Comunidad	234	69,9	35	66,1
Fuera de la comunidad	101	30,1	18	33,9
Atención hospitalaria				
En el municipio	184	54,9	30	56,6
Fuera del municipio	151	45,1	23	43,4
Acceso a programas de salud1				
Acceso hasta 5 programas	254	75,8	38	71,7
Acceso a más de 5 programas	80	23,9	15	28,3

Fuente: Elaboración propia. ESF/ NASF= Estrategia de Salud Familiar/Núcleo Ampliado de Salud Familiar. * Variables no excluyentes (puede responder más de una opción en una misma pregunta). ^1^En la variable acceso a programas de salud, en población estudiada, una persona no respondió.

Con relación al acceso a programas de salud, el 76% accede a un máximo de cinco programas, entre ellos: salud bucal (54,6%), vacunación (99,1%), salud de la mujer (61,1%) y farmacia popular (47,2%).

En cuanto a los programas específicos para poblaciones tradicionales, el 94,3% de las familias informó no tener acceso a ellos (Tabla 2). Esto podría deberse a la falta de reconocimiento de las identidades de estas poblaciones por parte del poder público, ya que muchas enfrentan dificultades en el proceso de reconocimiento formal. Estas dificultades se agravaron durante el período de 2019 a 2022, según los relatos de los propios participantes, confirmando la aceleración de políticas ultraconservadoras, de desprotección social, racistas y xenófobas en Brasil durante el gobierno de Jair Bolsonaro^25^.

Con relación a la asistencia psiquiátrica hospitalaria y en la Red de Atención Psicosocial, el 16,9% de los informantes de cada familia reportó tener algún familiar con historial de tratamiento en el centro de atención psicosocial, y solo el 8,3% reportó casos de internación hospitalaria. Es necesario considerar que estas comunidades tradicionales están ubicadas en municipios pequeños, en áreas rurales, lejos de los grandes centros urbanos en los que existe disponibilidad de camas psiquiátricas.

El estudio de Costa^26^ coincide con estos datos al señalar las dificultades que enfrentan las comunidades quilombolas para garantizar su acceso a los derechos básicos relacionados con políticas de infraestructura, como la falta de energía, agua y transporte. Esto termina comprometiendo el acceso pleno de estas comunidades a los servicios públicos. En línea con el estudio de Costa^26^, Oliveira^27^ destaca otro problema enfrentado por las comunidades quilombolas en el estado de Alagoas con respecto al acceso a los servicios públicos de saludo: o se encuentran lejos de los territorios quilombolas o, cuando están más cerca, presentan limitaciones en términos de inserción, aceptabilidad y diálogo con los saberes y prácticas asociados al modo de vida de sus residentes. Además, el hecho de que muchos problemas de salud mental ni siquiera sean mencionados por las personas de las comunidades a los equipos de APS y, por lo tanto, no son derivados a servicios de mayor complejidad puede ser un factor que influya en esta realidad^28^. Tratándose de comunidades tradicionales, “la forma de comprender y actuar en el mundo, vivida en el terrero, con sus mitos y ritos, sus creencias y valores, constituye un conjunto de saberes y verdades legítimas en su contexto y que, muchas veces, se contrapone y escapa a los saberes y verdades técnico-científicas de los profesionales”^29^ y del propio Sistema Único de Salud (SUS).

En cuanto al perfil de consumo de psicofármacos en las comunidades, la Tabla 3 presenta los datos principales. Los resultados obtenidos en términos del perfil de consumo de psicofármacos en comunidades indígenas y quilombolas de los cuatro estados investigados se alinean con otros estudios respecto a las clases más utilizadas^13^^,^^24^^,^^30^^,^^31^. Tal como muestra la Tabla 3, los antidepresivos ocuparon el primer lugar, seguidos por los ansiolíticos y antipsicóticos. Aunque en su mayoría eran prescritos por médicos, la ausencia de un seguimiento profesional durante el uso del medicamento y la falta de un plan terapéutico bien definido para el cuidado en salud mental, incluyendo la derivación a otros profesionales, es bastante significativa. Esto alerta sobre una realidad de automedicación, dependencia y cronificación entre los usuarios de estas comunidades^32^. En este contexto, la investigación reveló que el 43,4% de los usuarios no realizaba ningún tipo de seguimiento médico, mientras que el 42,3% informó haber interrumpido la farmacoterapia debido a molestias o efectos no deseados asociados a los psicofármacos, sin orientación médica, evidenciando una relación entre la adherencia a la farmacoterapia y el seguimiento profesional^33^.

**Tabla 3:** Perfil de utilización de psicofármacos en las comunidades indígenas y quilombolas. Estados de Minas Gerais, Rio Grande do Norte, Alagoas y Piauí, Brasil, marzo a junio de 2023.

Variables	Personas usuarias de psicofármacos (n=53)	
	n	%
Acceso a medicamentos*		
Establecimiento público	11	21,2
Farmacia	33	63,5
Acceso por terceros	1	1,9
Muestra gratis	1	1,9
**Prescripción de medicamentos**		
Prescrito por médico	49	92,5
No prescripto	4	7,5
**Tipo de prescriptor**		
Psiquiatra	37	48,1
Médico de atención primaria	31	40,3
Otra persona	9	11,7
**Acompañamiento médico**		
Sí	30	56,6
No	23	43,4
**Derivación hacia otro profesional**		
Sí	14	26,4
No	39	67,5
Clase de psicofármaco*		
Antidepresivo	40	75,5
Ansiolítico	18	34
Antipsicótico	6	11,3
Hipnótico/sedativo	4	7,5
Antiepiléptico	3	5,7
Estabilizadores del estado de ánimo	1	1,9
**Interrupción del uso de psicofármacos**		
Malestar o efecto indeseado	11	42,3
Dificultades para la obtención	3	11,5
Dificultades para recordar	2	7,7
Otro motivo	13	50
**Realizan psicoterapia**		
Sí	1	4,2
No	52	95,9

Fuente: Elaboración propia. * Variables no excluyentes (puede responder más de una opción en una misma pregunta).

En cuanto a la prescripción, los datos mostraron que el 92,5% de los usuarios de psicofármacos consumen medicamentos prescritos por médicos, mientras que el 7,5% usa medicamentos no prescritos, obtenidos sin receta. La automedicación es un problema frecuentemente reportado en investigaciones, a niveles más preocupantes que los encontrados en las comunidades de este estudio^34^^,^^35^. Sin embargo, el bajo nivel de automedicación podría estar relacionado con el perfil sociodemográfico y el lugar de residencia de esta población, que dificulta la obtención de psicofármacos^36^.

También se observó que el 48,1% de las prescripciones fueron realizadas por psiquiatras, mientras que el 40,3% provinieron de médicos del primer nivel de atención. Este resultado es coherente con la realidad de las comunidades que, en su mayoría, están ubicadas en zonas alejadas de los servicios especializados. Aunque una gran proporción de las prescripciones se obtiene en el primer nivel de atención, esto no garantiza un seguimiento sistemático, debido a la frecuente práctica de renovación de recetas mediante copias, es decir, la simple sustitución de recetas sin evaluación del estado clínico del usuario^37^^,^^38^.

Este es uno de los principales factores de inducción a la medicalización ya que, como señalan Bezerra *et al*., “el tratamiento de los trastornos mentales con psicofármacos es sintomático y su uso debe limitarse a lo imprescindible, considerando siempre si la relación riesgo-beneficio potencial del fármaco justifica su empleo y si otros recursos han sido debidamente explorados”^39^. La medicalización, como se describió anteriormente, está relacionada con la racionalidad tecnocrática del cuidado en salud, en la que la prescripción farmacológica y la producción de patrones de normalidad están en el centro del tratamiento. Esto compromete la autonomía de los usuarios, ya que genera una dependencia del consumo de psicofármacos de manera no planificada, sin una meta definida para el fin del tratamiento, haciendo que estos usuarios dependan del servicio para realizar su cuidado en salud mental^40^.

Otra consecuencia es el compromiso de la integralidad del cuidado, ya que los resultados indican la ausencia de un seguimiento multiprofesional, indispensable para un cuidado ampliado en salud. Como muestra la Tabla 3, entre las personas usuarias de psicofármacos, solo el 26,4% recibió orientación para la derivación a otro profesional, lo que muestra que el medicamento ha sido la opción prioritaria para el tratamiento de los problemas de salud mental. Solo una persona realizaba psicoterapia y la fuerte inversión en psicofármacos ocupa el espacio de otras prácticas terapéuticas, lo que denota un enfoque prescriptivo, biologicista y centrado en la medicina dentro del proceso de cuidado^41^.

De este modo, los resultados señalaron un perfil prescriptivo y verticalizado del servicio al que acceden estas comunidades, tal como describen otros estudios^42^, además de problemas en la gestión del cuidado, como la falta de seguimiento del uso de la medicación y la interrupción repentina y no asistida; prácticas que contradicen lo propuesto por las políticas de uso racional de medicamentos y los procesos de autogestión y corresponsabilidad del usuario, tal como lo promueve la atención psicosocial^43^.

En cuanto a la forma de acceso a los psicofármacos, se observa que el 63,5% de las personas que consumen medicamentos psicotrópicos los adquieren en farmacias mediante pago. Solo el 21,2% accedió a ellos de manera gratuita en establecimientos públicos (Tabla 3). Esta realidad también fue presentada en el estudio de Garcia *et al*.^44^, que señala que el gasto en medicamentos es el mayor gasto en salud entre las familias de menores ingresos. Además, según los autores, cuando se trata de medicamentos para el “sistema nervioso”, que incluyen todos los psicofármacos, esta es la tercera categoría más utilizada por familias brasileñas que enfrentan condiciones precarias de vivienda y alimentos insuficientes^44^. Así, el alto costo de los psicofármacos ciertamente impacta en el ya reducido presupuesto familiar y repercute en las condiciones generales de vida y en el acceso a otros bienes de consumo.

Entre los factores relacionados con la obtención o no de medicamentos en establecimientos públicos de salud, se destacan tres: la inclusión del medicamento en la Relación Nacional de Medicamentos Esenciales (RENAME), la disponibilidad de los medicamentos en la Red de Atención a la Salud y la facilidad de desplazamiento hasta el establecimiento público que realiza el dispendio de la medicación. En el presente estudio, se observó que, del total de 19 psicofármacos mencionados por las personas participantes, 10 no estaban incluidos en la RENAME (Tabla 4).

**Tabla 4:** Psicofármacos incluidos y no incluidos en RENAME, mencionados por las personas encuestadas en las comunidades indígenas y quilombolas. Estados de Minas Gerais, Rio Grande do Norte, Alagoas y Piauí, Brasil, marzo a junio de 2023.

Psicofármacos mencionados por las personas encuestadas	
Clonazepam	Alprazolam
Diazepam	Bromazepam
Amitriptilina	Desvenlafaxina
Fluoxetina	Duloxetina
Nortriptilina	Escitalopram
Gabapentina	Paroxetina
Clozapina	Sertralina
Olanzapina	Trazodona
Quetiapina	Pregabalina
	Zolpidem

Fuente: Elaboración propia.

Este resultado contradice las recomendaciones del Ministerio de Salud para la prescripción de medicamentos en el ámbito del SUS, que establece que se debe dar preferencia a los medicamentos incluidos en la RENAME como estrategia para mejorar el acceso y la adherencia al tratamiento, especialmente en poblaciones más vulnerables^45^. Lo que observamos es que más de la mitad de los medicamentos prescritos no estaba disponible en la red de salud, por lo tanto, solo eran accesibles mediante compra en farmacias, tal como se indicó anteriormente.

Es sabido que los estados y municipios brasileños cuentan con sus propias listas de medicamentos esenciales: la Relación Estatal de Medicamentos Esenciales (RESME) y la Relación Municipal de Medicamentos Esenciales (REMUME), respectivamente. Estas listas se basan en la RENAME y en el perfil fármaco-epidemiológico regional, orientando el alcance de medicamentos que deben ser adquiridos por estas entidades. Por lo tanto, las relaciones de medicamentos se fundamentan en criterios de costo-efectividad, costo-utilidad y costo-beneficio, evitando gastos innecesarios en la adquisición de medicamentos^45^.

Los datos obtenidos por la Pesquisa Nacional sobre Acesso, Utilização e Promoção do Uso Racional de Medicamentos (PNAUM), realizada entre 2014 y 2015, señalaron que el 55,2% del total de psicofármacos prescritos estaba incluido en la RENAME^2^, porcentaje similar al encontrado en el presente estudio. Esto podría indicar una similitud entre la tendencia nacional y la observada en las comunidades estudiadas. Sin embargo, la Organización Mundial de la Salud establece que lo ideal es que el 100% de los medicamentos prescritos para cualquier condición en los servicios públicos de salud pertenezcan a las listas de medicamentos esenciales, ya que esto garantiza el acceso a medicamentos con mejor relación costo-beneficio^46^.

Es necesario considerar, en el caso de las comunidades estudiadas, la distancia entre el lugar de residencia y el punto de dispendio de estos medicamentos. Frecuentemente, estos medicamentos se obtienen en municipios de mayor tamaño, polos regionales de salud, que suelen estar lejos de las comunidades rurales, lo que impacta negativamente en el acceso a través del sistema público de salud. Sin embargo, no se puede ignorar, como indica Preciado^47^, que los psicofármacos “se utilizan de manera ambivalente: tanto de forma estrictamente terapéutica como de manera política en los procesos de medicalización y regulación farmacológica de la existencia”. El mercado de los psicofármacos opera de forma insidiosa, atendiendo las expectativas de las personas usuarias respecto a la resolución de su malestar cotidiano, optimizando sus desempeños y posibilitando cierta normalización de los comportamientos y de la vida diaria.

Por otro lado, ayudan a los profesionales a resolver demandas psiquiátricas agudas y urgentes, pero especialmente en el manejo de situaciones cada vez más comunes, relacionadas con dificultades para dormir, vigilia, disposición y concentración, así como en el tratamiento de casos resistentes y refractarios que se configuran en nuevas cronicidades. Como señalan Pande y Amarante^48^:

…estos nuevos crónicos hacen uso recurrente, pero poco resolutivo, de los servicios de salud mental. Estos pacientes tienen dos características comunes: una severa dificultad en el funcionamiento social y la tendencia a usar los servicios de salud mental de forma inapropiada, drenando tiempo y energía de los clínicos, sin ajustarse completamente a un plan terapéutico.^48^

Además, facilitan la normalización de los comportamientos agitados de los pacientes psicóticos, promoviendo “la tranquilidad en las salas de los hospitales psiquiátricos”^49^

Otro aspecto evaluado en este estudio fue el uso de otros métodos y recursos para el cuidado autónomo. Considerando que los pueblos tradicionales suelen explorar recursos de hierbas medicinales para el cuidado de la salud^50^, se investigó el uso de compuestos vegetales, asociados o no al tratamiento con medicamentos.

Entre los 335 participantes del estudio, 80 reportaron utilizar algún tipo de compuesto vegetal (tés, jarabes, etc.) como recurso de autocuidado. De ese total, el 30% pertenece al grupo que utiliza psicofármacos y compuestos de manera combinada. Este es un dato preocupante debido a los riesgos de reacciones adversas o interacciones farmacológicas que puede generar la combinación de componentes de medicamentos industrializados con aquellos obtenidos de vegetales^51^. Además, las reacciones manifestadas por el uso conjunto de estas sustancias pueden provocar la discontinuidad del tratamiento medicamentoso o su uso inadecuado, dado que existe una escasez de orientación profesional^52^. Es conocido que las comunidades rurales tienen acceso limitado a los servicios de salud, especialmente a los de urgencia y emergencia. En caso de intoxicación aguda por el uso combinado de compuestos vegetales y medicamentos, la atención puede verse afectada. En el caso de intoxicaciones a largo plazo, el problema puede agravarse debido a la falta de seguimiento profesional, como ya se discutió en este estudio, dificultando la detección de los casos.

Tal como se muestra en la Tabla 5, entre las personas que usan psicofármacos (n=53), el 47,2% realizaba alguna actividad complementaria orientada al cuidado de la salud mental. En este grupo, se observó que la práctica religiosa era la más frecuente (56,5%). Los estudios indican que la asociación entre el tratamiento de salud mental y la práctica religiosa y/o espiritual produce efectos positivos, ya que la participación colectiva contribuye al aumento de la autoestima, la resiliencia, el empoderamiento del individuo y la resignificación de su sufrimiento. Por otro lado, en algunos casos, esta conexión puede resultar en una mayor carga de la enfermedad si está asociada a la explotación y manipulación mediante dogmas que generan culpabilidad, lo que puede conducir a una experiencia negativa para el sujeto^53^.

**Tabla 5:** Actividades complementarias de cuidado de la salud mental realizadas por las personas usuarias de psicofármacos en las comunidades indígenas y quilombolas. Estados de Minas Gerais, Rio Grande do Norte, Alagoas y Piauí, Brasil, marzo a junio de 2023.

Variables	Personas usuarias de psicofármacos (n=53)	
	n	%
Actividades complementarias		
Religiosa	13	56,5
Laboral	1	4,3
Física	4	17,4
Recreativa	5	21,7
Ninguna	28	52,5

Fuente: Elaboración propia.

Otro aspecto de la religiosidad a considerar es que, aunque esta dimensión es muy importante y constituye una parte esencial de las subjetividades, especialmente entre las poblaciones investigadas, los equipos de salud aún no están suficientemente capacitados para ofrecer prácticas integrativas y complementarias en salud que incluyan esta dimensión. Esto es particularmente relevante si consideramos que las personas con adherencia a prácticas religiosas/espirituales, como quilombolas e indígenas, tienen una probabilidad de reportar bienestar subjetivo un 5% mayor^54^, mientras que quienes se declaran poco espiritualizados tienen dos veces más probabilidades de presentar trastornos mentales y aproximadamente siete veces más posibilidades de recibir un diagnóstico de abuso o dependencia de alcohol^55^. Según Berni:

…la Organización Mundial de la Salud ha enfatizado la importancia de que los países miembros trabajen en la búsqueda de estrategias más efectivas para la promoción de la salud mental, incluidas la recuperación de prácticas tradicionales y técnicas complementarias, alternativas e integrativas basadas en el conocimiento.^56^

Las actividades recreativas representan el 21,7% (Tabla 5), un resultado que podría estar relacionado con la escasez de oferta de opciones de ocio para las poblaciones rurales, reflejo de la percepción social de que el medio rural está estrictamente vinculado a la producción agrícola^57^. Este es un resultado relevante, ya que estudiosos señalan que la falta de acceso a opciones de ocio es uno de los principales factores de riesgo para la aparición de trastornos mentales en comunidades rurales^58^, lo que puede considerarse un importante modulador de la prevalencia de sufrimiento psíquico en estas comunidades.

## CONCLUSIÓN

El objetivo fue analizar el perfil del uso de psicofármacos en comunidades indígenas y quilombolas de los estados de Minas Gerais, Rio Grande do Norte, Alagoas y Piauí en Brasil. Los psicofármacos más utilizados fueron los antidepresivos, seguidos por los ansiolíticos y antipsicóticos. Se identificó que el uso predomina entre mujeres adultas, con pocos años de escolarización, mayor edad, bajos ingresos y dependientes de programas sociales. En cuanto a la forma de acceso, pocos logran obtenerlos en establecimientos públicos de forma gratuita. Más de la mitad de los psicofármacos prescritos no están incluidos en la RENAME, lo que dificulta el acceso e impacta el presupuesto familiar debido a los altos costos. Aunque las prescripciones son realizadas por médicos, falta seguimiento profesional, y se detectaron casos de automedicación e interrupciones no planificadas. Este perfil señala claramente que las poblaciones tradicionales no tienen acceso universal a los servicios de salud, enfrentan múltiples barreras para acceder a los medicamentos, carecen de un seguimiento sistemático y un manejo adecuado de los casos de uso de psicofármacos, y también se ven afectadas por el grave problema de la automedicación presente en Brasil.

Este estudio destaca los impactos de las desigualdades sociales en la salud mental de poblaciones tradicionales históricamente vulnerabilizadas. El escenario observado en los cuatro estados refuerza la necesidad de ampliar las políticas públicas de desarrollo social en los territorios con poblaciones tradicionales, mejorando sus condiciones de vida, pero especialmente las políticas de salud mental dirigidas a las poblaciones indígenas y quilombolas, con énfasis en la atención primaria, una realidad aún muy incipiente a nivel nacional.

La integralidad del cuidado en salud mental no se limita a la oferta de alternativas farmacológicas para el tratamiento de problemas de salud mental, sino que depende de un conjunto de medidas destinadas a mejorar las condiciones de vida de las poblaciones tradicionales, reducir las desigualdades sociales, ampliar el acceso a los servicios de salud y ofrecer cuidados culturalmente adecuados, respetando la diversidad cultural existente y comprendiendo que sus necesidades de salud mental están estrechamente relacionadas con las disparidades socioeconómicas que se traducen en precariedad de vida^59^.

En este sentido, resulta urgente realizar más estudios sobre la salud mental y el uso de psicofármacos en poblaciones quilombolas e indígenas, con el fin de proponer reflexiones epistemológicas y diálogos interculturales orientados a la salud mental que favorezcan el desarrollo de una “escucha cualificada del sufrimiento/aflicción, en una clínica redimensionada por un perspectivismo cultural”^9^.

## References

[1] Barros JC, Silva SN (2023). Perfil de utilização de psicofármacos durante a pandemia de COVID-19 em Minas Gerais, Brasil. Revista Brasileira de Epidemiologia.

[2] Brasil, Ministério da Saúde, Secretaria de Ciência, Tecnologia e Insumos Estratégicos (2016). Pesquisa Nacional sobre Acesso, Utilização e Promoção do Uso Racional de Medicamentos no Brasil, Componente populacional: resultados.

[3] Boni BS, Rezende KTA, Mazzeto FMC, Tonhom SFR, Rezende M (2021). The use of psychiatric drugs: an integrative review. New Trends in Qualitative Research.

[4] Oliveira JRF, Varalho FR, Jirón M, Ferreira IML, Siani-Morello MR, Lopes VD (2021). Descrição do consumo de psicofármacos na atenção primária à saúde de Ribeirão Preto, São Paulo, Brasil. Cadernos de Saúde Pública.

[5] Costa EAP, Galvão DO, Figueiredo CFS, Rodrigues TA, Neto LJ (2023). Uso indiscriminado de psicotrópicos por usuários assistidos na atenção primária a saúde: uma revisão integrativa da literatura. Humanum Sciences.

[6] Secco AC, Tesses CD (2023). Revisitando Whitaker: psicofármacos e cuidado em Saúde Mental na Atenção Primárias à Saúde. Saúde em Debate.

[7] Oliveira J, Cavalcanti F, Ericson S (2024). Medicalização da subjetividade e fetichismo psicofármaco: uma análise dos fundamentos. Saúde e Sociedad.

[8] Pizzinato A, Guimarães DS, Leite JF (2019). Psicologia, povos e comunidades tradicionais e diversidade etnocultural. Psicologia: Ciência e Profissão.

[9] Batista MQ, Zanello V (2016). Saúde Mental em contextos indígenas: escassez de pesquisas brasileiras, invisibilidade das diferenças. Estudos de Psicologia.

[10] Noal DS, Rabelo IVM, Chachamovich E (2019). O impacto na saúde mental dos afetos apóes o rompimento da barragem da Vale. Cadernos de Saúde Pública.

[11] Ferreira JC (2022). Impactos da COVID-19 nas territorialidades tradicionais quilombolas do município de Bom Conselho - Pernambuco. Revista Contexto Geográfico.

[12] Werneck GL (2022). A pandemia de COVID-19: desafios na avaliação impacto de problemas complexos e multidimensionais na saúde de populações. Cadernos de Saúde Pública.

[13] Barbosa VFB Cabral LB, Alexandre ACS (2019). Medicalização e saúde indígena: uma análise do consumo de psicotrópicos pelos índios Xukuru de Cimbres. Ciência e Saúde Coletiva.

[14] Barreto IF, Dimenstein M, Leite JF (2022). Percepções sobre o uso de álcool em uma comunidade indígena potiguar. Psicologia: Teoría e Pesquisa.

[15] Loyola AI, Firmo JOA, Mambrini JVM, Peixoto SV, Souza PRB, Mambrini JVM (2022). Uso de psicofármacos por população em área atingida pelo rompimento de barragem de rejeitos: Projeto Saúde Brumadinho. Revista Brasileira de Epidemiologia.

[16] Dimenstein M, Belarmino VH, Martins ME, Dantas C, Macedo JPS, Leite JF (2020). Desigualdades, racismos e saúde metnal em uma comunidade quilombola rural. Amazônica, Revista de Antropologia.

[17] Dimenstein M, Belarmino VH, Leite JF, Macedo JPS, Silva IT, Dantas C (2019). Consumo de álcool em uma comunidade quilombola do nordeste brasileiro. Quaderns de Psicología.

[18] Xavier LC, Batista LE, Wernek J, Lopes F (2012). Saúde da população negra.

[19] Monego ET, Peixoto MRG, Cordeiro MM, Costa RM (2010). (In)segurança alimentar de comunidades quilombolas do Tocantins. Segurança Alimentar e Nutricional.

[20] Batista EC, Rocha KB (2020). Saúde Mental em comunidades quilombolas do Brasil: uma revisão sistemática da literatura. Interações (Campo Grande).

[21] Capp E, Nienov OH (2021). Epidemiologia aplicada básica.

[22] Morisky DE, Green LW, Levine DM (1986). Concurrent predictive validity of a self-reported measure of medication adherence. National Library of Medicine.

[23] Fontanella AT (2017). Uso de psicofármacos: uma abordagem de gênero: dados da pesquisa nacional sobre o acesso, utilização e promoção do uso racional de medicamentos - PNAUM 2014.

[24] Siani-Morello MR, Pereira LB, Ferreira IML, Aliste MJ, Pereira LRL (2022). Psychoactive drugs in the Brazilian public health system: use profile and associated factors. Brazilian Journal of Pharmaceutical Sciences.

[25] Castilho DR, Lemos ELS (2021). Necropolítica e governo Jair Bolsonaro: repercussões na seguridade social brasileira. Revista Katálysis.

[26] Costa ES (2012). Racismo, política pública e modos de subjetivação em um quilombo do Vale do Ribeira.

[27] Oliveira JR, Riscado JLS, Oliveira MAB (2011). Quilombolas, guerreiros alagoanos: AIDS, prevenção e vulnerabilidade.

[28] Lopes MO, Soares TCM, Bezerra STF (2023). Ressignificando o papel da Atenção Primária como porta de entrada para oferta do serviço e organização do território. Espaço para a Saúde.

[29] Alves MC, Semiotti N (2009). Atenção à saúde em uma comunidade tradicional de terreiro. Revista de Saúde Pública.

[30] Araújo AFLL, Ribeiro MC, Vanderlei AD (2021). Automedicação de psicofármacos entre estudantes universitários de odontologia e medicina. Revista Internacional de Educação Superior.

[31] Zuanazzi CA, Grazziotin NA (2020). Análise da dispensação de antidepressivos e ansiolíticos em uma farmácia comercial do Noroeste do Rio Grande do Sul. Perspectiva.

[32] Almeida LM, Fernandes WOB, Ferreira EMR (2021). Uso abusivo de psicofármacos e o papel do farmacêutico na prevenção da medicalização. Revista Saúde & Ciência Online.

[33] Silva SN, Lima MG (2017). Assistência Farmacêutica na Saúde Mental: um diagnóstico dos Centros de Atenção Psicossocial. Ciência & Saúde Coletiva.

[34] Xavier MS, Castro HN, Souza LGD, Oliveira YSL, Tafuri NF, Amâncio NFG (2021). Automedicação e o risco à saúde: uma revisão de literatura. Brazilian Journal of Health Review.

[35] Gonçalves J, Moura SES, Dantas GCL, Lima AM, Brito WSB, Siebra BOB (2018). Influência da publicidade na automedicação na população de um município brasileiro de médio porte. Journal of Health and Biological Sciences.

[36] Silva IDD, Bezerra INM, Pimenta IDSF, Silva G, Wanderley VB, Nunes VMA (2019). Acesso e implicações da automedicação em idosos na atenção primária à saúde. Journal Health NPEPS.

[37] Silveira ER (2012). Práticas que integram a saúde mental à saúde pública: o apoio matricial e interconsulta. Ciência & Saúde Coletiva.

[38] Matoso KFC, Moura PC (2018). O uso indiscriminado de benzodiazepínicos por idosos atendidos na atenção primária de Fexlância, Minas Gerais. Revista Brasileira de Ciências da Vida.

[39] Bezerra IC, Morais JB, Paula ML, Silva TMR, Jorge MSB (2016). Uso de psicofármacos na atenção psicossocial: uma análise à luz da gestão do cuidado. Saúde em Debate.

[40] Zanella CG, Aguiar PM, Storpirtis S (2015). Atuação do farmacêutico na dispensação de medicamentos em Centros de Atenção Psicossocial Adulto no município de São Paulo, SP, Brasil. Ciência & Saúde Coletiva.

[41] Peralta PEC, Nascimento AKC, Santiago E (2023). Medicalização, saúde mental e biopolítica: uma revisão integrativa. Saúde e Pesquisa.

[42] Merhy EE, Onocko-Campos RT (1997). Agir em Saúde: um desafio para o público.

[43] Colaço RF, Onocko-Campos RT (2022). Gestão compartilhada do tratamento com psicofármacos: inquérito com usuários de CAPS de quatro grandes cidades brasileiras. Ciência & Saúde Coletiva.

[44] Garcia LP, Sant’Anna AC, Magalhães LCG, Freitas LRS, Aurea AP (2013). Gastos das famílias brasileiras com medicamentos segundo a renda familiar: análise da Pesquisa de Orçamentos Familiares de 2002-2003 e de 2008-2009. Cadernos de Saúde Pública.

[45] Brasil, Ministério da Saúde (2020). Proposta de método ativo de atualização da RENAME: Parte 1 - Guia para identificação sistemática de necessidades de análise do elenco da atenção primária à saúde.

[46] Organização Mundial da Saúde (2002). Perspectivas sobre a Política de Medicamentos: A seleção de medicamentos essenciais.

[47] Preciado PB (2018). Texto Junkie: sexo, drogas e biopolítica na era farmacopornográfica.

[48] Pande MNR, Amarante PDC (2011). Desafios para o centros de atenção psicossocial como serviços substitutivos: a nova cronicidade em questão. Ciência & Saúde Coletiva.

[49] Caponi S (2022). O mercado dos psicofármacos e a gestão das emoções em tempos pós pandémicos. Política & Sociedade.

[50] Castro MR, Figueiredo FF (2019). Saberes tradicionais, biodiversidade, prática integrativas e complementares: o uso de plantas medicinais no SUS. Hygeia - Revista Brasileira de Geografia Médica e da Saúde.

[51] Gonçalves RN, Gonçalves JRSN, Buffon MCM, Negrelle RRB, Rattmann YD (2022). Plantas medicinais na atenção primária à saúde: riscos, toxicidade e potencial para interação medicamentosa. Revista de APS.

[52] Silva MC, Colino OS, Pontes JG (2021). Interações medicamentosa em fitoterápicos. Research, Society and Development.

[53] Oliveira MR, Junges JR (2012). Saúde mental e espiritualidade/religiosidade: a visão de psicólogos. Estudos de Psicologia.

[54] Souza LDM, Maragalhoni TC, Quincoses MT, Jansen K, Cruzeiro ALS, Ores L (2012). Bem-estar psicológico de jovens de 18 a 24 anos: fatores associados. Cadernos de Saúde Pública.

[55] Gonçalves JS, Silva LL, Abdala GA, Meira MDD, Santos ACM, Silva MFF (2017). Religiosity and common mental disordes in adults. Revista de Enfermagem UFPE Online.

[56] Berni LEV (2017). Psicologia e saúde mental indígena: um panorama para construção de políticas públicas. Psicologia para América Latina.

[57] Maziero C, Godoy CMT, Campos JRR, Mello NA (2019). O lazer como fator de permanência e reprodução social no meio rural: estudo do município de Saudade do Iguaçu, PR. Interações (Campo Grande).

[58] Morais SRS, Sena L, Baldo AM, Souto BS (2020). Saúde mental em territórios rurais: a experiência do PET-Saúde GraduaSUS UNIVASF. Extramuros - Revista de Extensão da UNIVASF.

[59] Dantas MB, Dimenstein M, Leite JF, Macedo JP, Belarmino VH (2020). Território e Determinação Social da Saúde Mental em contextos rurais: cuidado integral às populações do campo. Athenea Digital.

